# Single Cell Transfection through Precise Microinjection with Quantitatively Controlled Injection Volumes

**DOI:** 10.1038/srep24127

**Published:** 2016-04-12

**Authors:** Yu Ting Chow, Shuxun Chen, Ran Wang, Chichi Liu, Chi-wing Kong, Ronald A. Li, Shuk Han Cheng, Dong Sun

**Affiliations:** 1 Department of Mechanical and Biomedical Engineering, City University of Hong Kong, Hong Kong, China; 2 Department of Biomedical Science, City University of Hong Kong, Hong Kong, China; 3 Stem Cell and Regenerative Medicine Consortium, Department of Physiology, LKS Faculty of Medicine, University of Hong Kong, Hong Kong, China

## Abstract

Cell transfection is a technique wherein foreign genetic molecules are delivered into cells. To elucidate distinct responses during cell genetic modification, methods to achieve transfection at the single-cell level are of great value. Herein, we developed an automated micropipette-based quantitative microinjection technology that can deliver precise amounts of materials into cells. The developed microinjection system achieved precise single-cell microinjection by pre-patterning cells in an array and controlling the amount of substance delivered based on injection pressure and time. The precision of the proposed injection technique was examined by comparing the fluorescence intensities of fluorescent dye droplets with a standard concentration and water droplets with a known injection amount of the dye in oil. Injection of synthetic modified mRNA (modRNA) encoding green fluorescence proteins or a cocktail of plasmids encoding green and red fluorescence proteins into human foreskin fibroblast cells demonstrated that the resulting green fluorescence intensity or green/red fluorescence intensity ratio were well correlated with the amount of genetic material injected into the cells. Single-cell transfection via the developed microinjection technique will be of particular use in cases where cell transfection is challenging and genetically modified of selected cells are desired.

Cell transfection is a process wherein foreign genetic substances (e.g., nuclei acids) are delivered into cells; this technique has been widely used to produce genetically modified cells. Genetic materials or gene products are usually delivered to enhance or inhibit specific gene expressions in cells so that the functions of the genes of interest could be studied^1^. The current methods of cell transfection involve bulk gene transfection, which requires large copy numbers of an expression vector per cell and relies on a stochastic process to deliver an average dose. Because the function of a cell is determined by its location and time, single-cell resolution of gene expression is important to elucidate gene functions. Thus, the design of a highly quantitative method to deliver precise amount of genetic modification substance into single cells is necessary.

Cell transfection methods may be broadly classified as biological, chemical, or physical methods^1^. Biological methods employ viral vectors to achieve gene transfer^2^, but they suffer from safety issues, such as promotion of immune responses or genetic mutations^3^. Chemical methods, such as lipofection reagents^4^,^5^, present the advantages of safety, large-scale production, and capacity to deliver large gene fragments. However, the transfection efficiency of these methods is largely affected by the cell type, reagent formulation, and DNA/reagent ratio, among others^6^. Physical methods can achieve bulk transfection or single cell transfection by utilizing diverse physical tools for delivering genetic substances. Some physical methods, such as ultrasound-based sonoporation^7^, magnetic field-based magnetofection^8^, and electric field-based electroporation^9^, can transfect a large number of cells by creating transient holes in the cell membrane to allow nucleic acids passage. Other methods using laser irradiation^10^, mechanical constriction^11^, or micropipette penetration^12^ can also achieve transfection for specific cell types or subcellular regions. However, although most physical methods can achieve specific delivery, they still rely on stochastic processes to deliver an average dosage and suffer from relatively low controllability of the amount of substance delivered.

Microinjection, a process of biological material delivery by insertion of a micropipette into living cells in culture, has been applied to many biomedical applications^12^,^13^,^14^,^15^, such as direct injection of nucleic acids into the cytoplasm or nucleus. Microinjection presents unique advantages during single-cell transfection, including cost savings through control of the amount of injected material delivered, applicability to different cell types and injection substances, and enhanced safety by virtue of its virus-free nature. Several microinjection systems have been integrated with robotics technology to enable automated injection with high transfection efficiency^16^,^17^,^18^,^19^,^20^. Most microinjection approaches focus on automating the injection process to overcome several problems that inherently exist during manual operation^21^, such as human fatigue and poor reproducibility. Only a few of these approaches have investigated single-cell transfection, which requires highly quantitative control of the delivered substance. Although manual quantitative microinjection has been applied to large-sized mouse embryos and zygotes to evaluate the effects of chemical compounds on embryo development^22^, delivery was neither automated nor reproducible and the process could not be applied to human cells, which usually range in size from 7 μm to 25 μm. To create genetically modified cells with predictable functions, a reliable and high-throughput quantitative microinjection technique that allows precise delivery of small amount of injection materials into a batch of human cells must be applied.

This article presents a new quantitatively controlled microinjection technique to achieve single-cell transfection. Based on an automated micropipette-based microinjection platform^23^ that employs a microfluidic chip to pattern the suspended cells in an array for easy injection and measurement, a technology that could achieve precise delivery of controlled amounts of materials into cells was developed. Specifically, the injection volume was measured by volumetric changes in water droplets dispensed in mineral oil to permit measurable injections under different injection pressures and times. To examine the accuracy of the delivered volume, water droplets with sizes similar to that of human cells were injected with fluorescent dye under calibrated injection parameters. Thereafter, the injection amount was calculated from the fluorescence intensity of the droplets, and the results verified the effectiveness of the injection volume control technique in delivering small amounts of a substance into cells. Experiments wherein different amounts of synthetic modified mRNA (modRNA) or a cocktail of plasmids were delivered into human foreskin fibroblast (HFF) cells at different concentration ratios were then performed to demonstrate the effective control of the materials delivered. All of the experimental results confirmed that the proposed quantitative microinjection technique can be readily applied to achieve precise single-cell transfection.

## Results

### Injection volume calibration results

**Figure 1:**
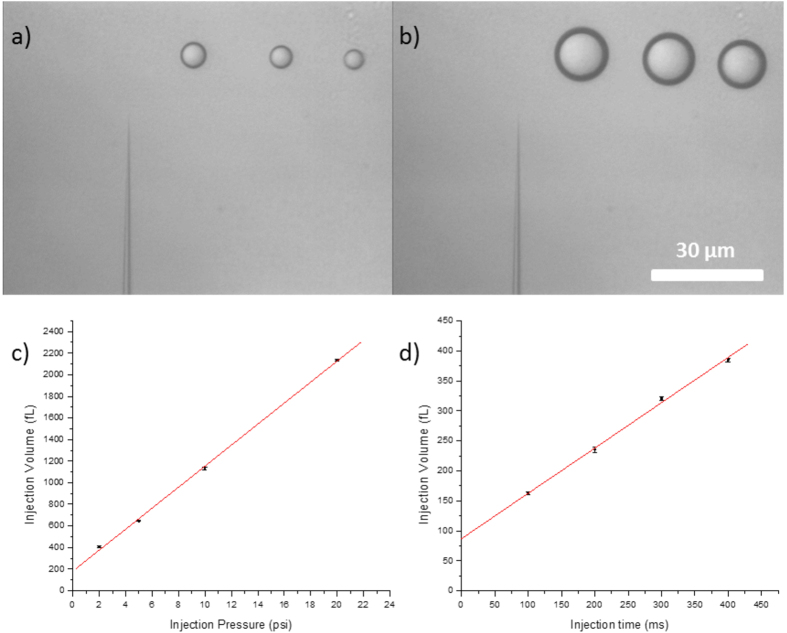
Injection amount calibration. (**a**) Water droplets dispensed in oil. (**b**) Water droplets after injection. Scale bar, 30 μm. (**c**) Linearity of injection volume versus injection pressure (R^2^ = 0.999, y = 181.1 + 97.3x). (**d**) Linearity of injection volume versus injection time (R^2^ = 0.999, y = 86.9 + 0.756x). Error bars in (**c**,**d**) indicate the SD of three independent experiments.

Control of the injection volume is achieved by adjusting the injection pressure and time. The micropipette was first calibrated with different injection parameters, and the injection volume was measured by examining the volumetric change in water droplets before and after microinjection. While Fig. 1a shows the water droplets dispensed in mineral oil before injection, Fig. 1b shows that the volume of the droplets increased after 1–30 injections. The computation results in Fig. 1c show the linear increase in the volume of the droplets with increasing injection pressure for an injection time of 100 ms. Figure 1d illustrates the linear increase in the volume of the water droplet with increasing injecting time under an injection pressure of 21.4 kPa. In the following cell injection experiments, the injection time was maintained at 100 ms, and the applied pressure was adjusted to achieve the desired injection volume. The operation process was computer-controlled to ensure that it is repeatable and consistent.

### Pseudo cell injection results

**Figure 2:**
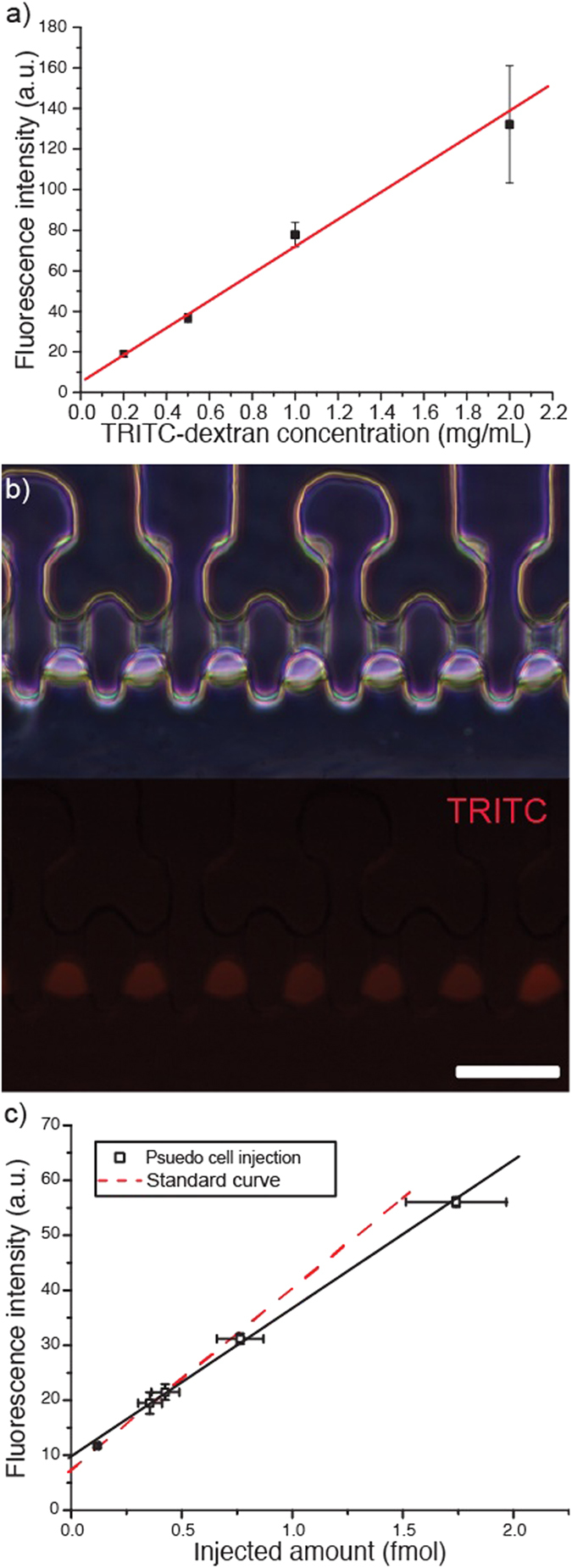
Pseudo Cell injection. (**a**) Fluorescence intensity as a function of TRITC-dextran concentration (R^2^ = 0.993, y = 5.05 + 66.9x, n = 5). (**b**) Droplets after TRITC-dextran injection. Scale bar, 50 μm. (**c**) Fluorescence intensity of pseudo cells after quantitative microinjection (R^2^ = 0.999, y = 9.66 + 26.9x).

To verify the accuracy of the calibration results, tetramethylrhodamine isothiocyanate (TRITC)-dextran was injected into water droplets dispensed in the cell trapping channel of a cell holder chip. Figure 2a illustrates variations in fluorescence intensity as a function of the TRITC-dextran concentration; a strong linear correlation between the measured fluorescence intensity and the TRITC-dextran concentration could be observed. The fluorescence intensity–TRITC concentration curve was obtained by dispensing a known concentration of TRITC-dextran solution in oil to form TRITC-dextran droplets and then measuring the resulting fluorescence intensity of each TRITC-dextran droplet. This curve is applied in this work as a standard curve to calculate TRITC-dextran concentration within water droplets after quantitative microinjection.

The injected water droplets, as shown in Fig. 2b, revealed similar fluorescence intensities (SD-to-Mean ratio = 0.124, n = 26), indicating that the precise injection volume is reproducible. Figure 2c shows that the fluorescence intensity curve obtained based on increasing injection amounts of TRITC-dextran was linear and similar to the standard curve, subject to a maximum error of 20.3%. Similar result was obtained after TRICT-dextran was injected into HFF cells, as seen in Supplementary Fig. 1. This result verifies that the injection system is capable to deliver accurate volume to target cells.

### modRNA injection results

**Figure 3:**
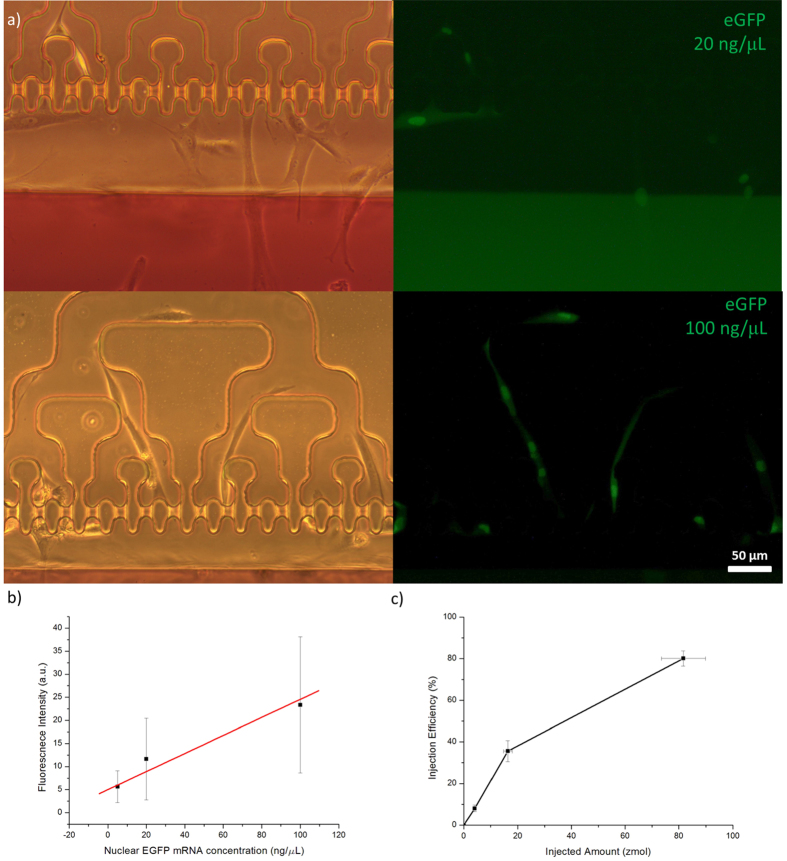
Delivery of Different Concentrations of modRNA encoding Nuclear eGFP. (**a**) HFF cells injected with different concentrations of nuclear eGFP encoding modRNA. (**b**) Linear change in fluorescence intensity with increasing amount of injected modRNA (R^2^ = 0.966, y = 5.03 + 0.195x). Each measurement represents the mean ± SD of at least 46 cells. (**c**) Transfection efficiency of injection of different concentrations of modRNA. Each measurement represents the mean ± SD of at least 3 independent experiments.

Quantitative injection of modRNA encoding nuclear eGFP into HFF cells was performed. About 420 fL of modRNA of different concentrations (5, 20, and 100 ng/μL) were separately injected into HFF cells. Fluorescence signals were checked 18 h after injection. Figure 3a shows HFF cells with different eGFP intensities, which indicates that the cells received different amount of modRNA. As the amount of the injected modRNA increased, the HFF cells showed increasing eGFP intensities, which means more eGFP protein had been synthesized in the cell. Figure 3b shows the linear change in fluorescence intensity with increasing injection amount of the modRNA. The amount of mRNA can determine the protein expression level inside the cell, and our results indicated that the amount of synthesized protein inside the living cells could be controlled by quantitative microinjection of the modRNA. We further measured the transfection efficiency of delivering different amounts of modRNA to the HFF cells. The transfection efficiency is defined as the ratio of the number of cells with nuclear eGFP to the number of injected cells. Figure 3c shows that the transfection efficiency was enhanced as the injected amount of modRNA increased. Transfection efficiency reached 80% when 100 ng/μL modRNA was injected into the cells. Since our previous experiment on TRITC-dextran injection of HFF cells showed a maximum injection efficiency of 88%^23^, further increases in modRNA injection amount may not show much increases in transfection efficiency.

Cell viability was investigated by incubating the cells in culture medium with 0.5 μM of SYTOX Orange dye (Molecular Probes) for 10 min. Supplementary Fig. 2a,b show the trapped cells in the cell holder chip without injection after 1 h incubation. It was found that 97.5% ± 2.0% (mean ± SD, n = 3) trapped cells were viable and adhered to cell holder chip. Supplementary Fig. 2c,d show the fluorescein isothiocyanate (FITC)-dextran injected cells in the cell holder after 1 h incubation. The viability of FITC-dextran injected cells could reach 82.1% ± 7.0% (mean ± SD, n = 3) examined by SYTOX Orange dye.

### Plasmid cocktail injection results

**Figure 4:**
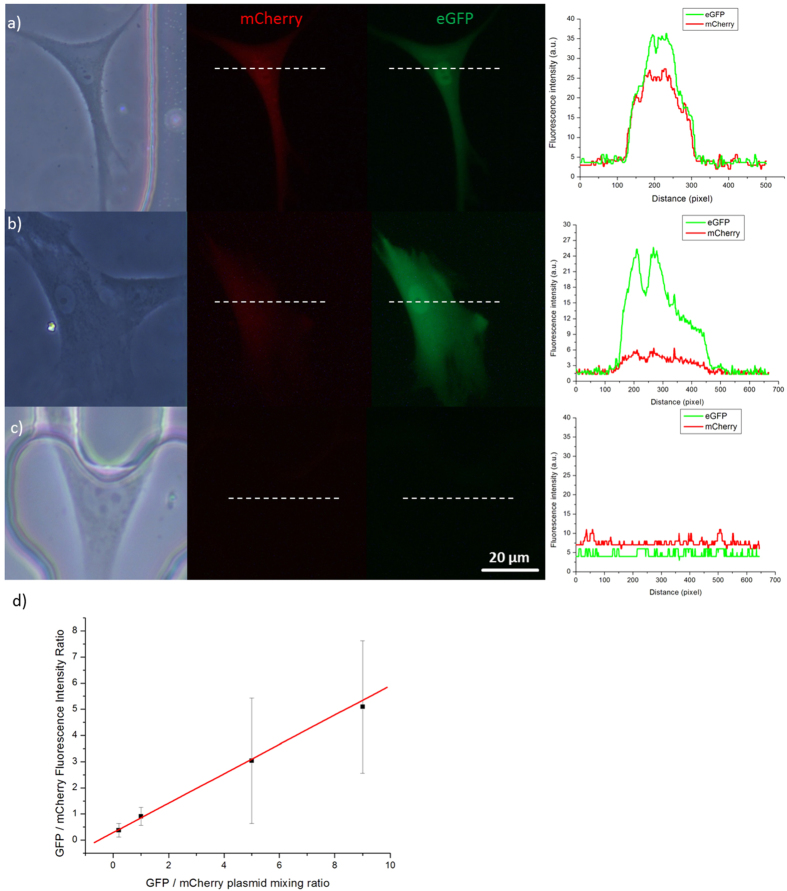
eGFP and mCherry Plasmid Cocktail Injection. **(a**) HFF cell injected with a mixture of eGFP and mCherry plasmids at a 1:1 ratio. (**b**) HFF cells injected with a mixture of eGFP and mCherry plasmids at a 9:1 ratio. (**c**) HFF cell without injection. Scale bar, 20 μm. (**d**) Ratio of eGFP/mCherry fluorescence intensity in relation to the ratio of the eGPF/mCherry plasmids (R^2^  = 0.998, y = 0.368 + 0.514x). Each measurement represents the mean ± SD of at least 50 cells.

DNA delivery into cells is also an important strategy in many cell biological studies. Cellular heterogeneity presents different cell behaviors among cells and plays a crucial role in studies of single-cell biology. We performed 14 plasmid cocktail injection experiments; in each experiment, a mixture of different ratios of plasmids encoding eGFP and plasmids encoding mCherry were injected into HFF cells. Figure 4a,b show the HFF cells 48 h after injection of eGFP and mCherry plasmids at ratios of 1:1 and 9:1, respectively. In these experiments, 11.8% ± 0.807% (mean ± SD) of the positive cells showed single eGFP expression, 4.10% ± 0.68% (mean ± SD) of the positive cells showed single mCherry expression, and 82.8% ± 2.99% (mean ± SD) of the positive cells showed co-expression of both eGFP and mCherry. The intensities of eGFP and mCherry were correlated with their injected plasmid concentrations in the cell. Figure 4c shows that the intensity ratio between eGFP and mCherry presents a linear relationship with the injected plasmid ratio. These results indicate that the ratio of synthesized target protein could be controlled by quantitatively injecting plasmids of an appropriate ratio into single cells.

## Discussion

In this study, we demonstrated single-cell transfection by using a precise micropipette-based microinjection technology with a quantitatively controlled injection volume. The delivered amount of injected substance can be quantified in terms of injection pressure and time, as evidenced by our calibration study where the injection volume was measured through volumetric changes in water droplets in mineral oil. The pseudo cell injection experiments verified that the proposed approach can achieve precise delivery of a small amount of fluorescent dye into target droplets, and the delivery amount and fluorescence intensity were linearly correlated. Compared with other transfection methods, such as electroporation and lipofection, the proposed method can potentially accomplish single-cell transfection regardless of the cell type or substance delivered. The effectiveness of the proposed technology was further demonstrated by injecting modRNA and a plasmid cocktail into HFF cells. Results agreed well with our hypothesis that higher concentrations of modRNA delivered can result in higher transfection efficiency before saturation is reached, thereby implying that the amount of substance delivered into the cells is a key factor in improving modRNA transfection efficiency. Our technology also allows microinjection of multiple components into a single cell. Experimental results of plasmid cocktail delivery into HFF cells verified that plasmid mixtures with different ratios of eGFP and mCherry could affect the amount of protein synthesized.

The proposed quantitative microinjection technique can be applied to study cell responses to different dosage of exogenous DNA or RNA. In general, modRNA delivery presents several advantages over plasmid DNA delivery in gene therapy applications^24^. Synthetic modified mRNA (modRNA) is a novel reprogramming tool generally used for controlled intracellular targeting and *in situ* logic evaluation of disease-related conditions^25^. The proposed method allows elucidation of single-cell gene expression in terms of number of RNA complexes delivered and number of proteins expressed through precise control of the delivery of modRNA. Moreover, RNA interference is steadily becoming a powerful tool to knockdown specific genes^26^. Our technique can serve as a more effective and reliable tool to deliver small inhibitory RNAs (siRNAs) in clinical applications. Yet another potential application of this method is injection of CRISPR/CAS9 systems for multiplex genome editing^27^. Delivery of CRISPR/CAS9 systems containing multiple genes of interest into cells could generate models for genetic disease studies^28^. Since the CRISPR/CAS9 system consists of multiple components and tight control of the dose and duration of CRISPR/CAS9 expression is critical for tuning targeting specificity^29^, the proposed quantitative microinjection technology may present an ideal solution through which both donor plasmid and CRISPR/CAS9 plasmid delivery into cells may be simultaneously accomplished to increase gene knock-in efficiency.

The proposed approach can be applicable across wide range of cell types, especially those with difficulty in treatment with traditional methods. The microinjection system can function as an enabling research tool for its ability of delivering virus-free DNA and RNA to transfect those challenged primary cells such as post-mitotic neurons^30^,^31^ and mesenchymal stem cells^32^. Through further advancement of microfluidic-based cell holder chip, high-throughput and multi-treatment modules can be further achieved.

## Materials and Methods

### Microinjection system setup

The microinjection experiments were conducted on an automated microinjection system as previously reported^23^. In brief, the system consisted of a 3-DOF robot manipulator to hold the injection pipette and cell holder chip, an objective lens equipped with a CCD camera for visual feedback, a microinjector to provide a positive injection pressure and a negative cell trapping pressure, and a PC with motion control for full automation programming. The vacuum-based cell holder chip with a two-layer structure of 256 cell trapping channels (See supplementary Fig. 3) can pattern cells on the array within 10 min. The optimal dimension of the trapping channel depended on the size of the targeted cells. The height of the trapping channel should be the same as or slightly smaller than the diameter of the target cell, while the thickness of supporting layer (see supplementary Fig. 3c) should be at least 4 times smaller than the diameter of the target cell.

The cell holder chip was fabricated by soft lithography replica molding technology with polydimethylsiloxane (PDMS, SYLGARD). Briefly, two UV masks with feature of each layer were printed by a high-resolution printer. The mold was fabricated by patterning a photoresist (GM1050, Gersteltec Sàrl) on a silicon wafer using the UV-mask. Following post-exposure baking, the second UV mask was aligned precisely with the first mask layer using a mask aligner (MA6, Karl Suss). The photoresist was then developed to remove all unexposed portions and create a permanent mold. PDMS molding was carried out to obtain the reverse structure of the master. The fully cured PDMS was peeled off from the master and trimmed under a microscope with a 5 × objective (Mitutoyo, Japan). An outlet was punched through the PDMS using a sharpened syringe needle, and the trimmed chip was bonded to a cover glass surface using plasma cleaner (PDC-002, Harrick Plasma).

### Cell culture

HFF cells were maintained in Dulbecco’s modified Eagle’s medium (Gibco) supplemented with 10% fetal bovine serum (FBS, Gibco), 100 U/mL penicillin, and 100 U/mL streptomycin (Antibiotic-Antimycotic, Gibco) in a humidified atmosphere of 37 °C and 5% CO_2_.

### Calibration

Volumetric changes in the droplets were used to characterize the injection volume of the micropipette. The micropipettes were routinely fabricated from glass capillary tubes (BF100-50-15, Sutter Instruments) using a programmable laser-base pipette puller (P-2000, Sutter Instruments). The tip diameter of the fabricated micropipette was measured by scanning electron microscopy, and micropipettes with a tip inner diameter of 0.5 μm were selected. After back filling with 1 μL of deionized water, the micropipette was fixed to the microrobot and connected to the microinjector, which controls the injection pressure and time. The pipette tip was then immersed into the mineral oil and water droplets were dispensed by adjusting the injection pressure or time. Since the droplets formed were very small, multiple injections were required to produce visible size changes in the droplets. Droplets were assumed to be spherical, and their volumes were determined by measuring the area of each droplet from captured images (See Supplementary Fig. 4a). The amount of injected substance could be determined by quantitatively injecting a certain volume of a substance with known concentration. Once the calibration results of injection volume versus injection pressure and time were obtained, the calibrated micropipette could be used for cell injection.

### Pseudo cell injection

Before pseudo cell injection, a standard fluorescence curve of TRITC-dextran was obtained by dispensing different concentration of TRITC droplets in oil and measuring the fluorescence intensity of the droplets. This curve was used to calculate the TRITC-dextran concentration for a given injected droplet.

For pseudo cell injection, negative pressure was applied to the outlet of the cell holder chip immersed in mineral oil. Each cell-trapping channel was inspected and all bubbles were removed. To create pseudo cells, the micropipette was filled with 1 μL of deionized water and inserted into the open end of a cell trapping channel. A 20 μm-diameter water droplet was dispensed into each channel. After their creation, pseudo cells were injected with 3 mg/mL TRITC-dextran using a calibrated micropipette under predefined injection parameters. The intensity of injected water droplets was measured and compared to that calculated from the standard curve based on TRITC-dextran concentration.

### modRNA injection

The procedure of automated cell injection experiments is descripted in supplementary Fig. 4b. Prior to the injection experiments, single HFF cells obtained by enzymatic dissociation were rinsed three times with PBS and then suspended in the culture medium. The cell solution was gently pipetted up and down and then filtered through a 40 μm cell strainer to prevent blocking of the cell trapping channels by large cell clusters. The sterilized cell holder chip was placed in a 35 mm culture dish, connected to the negative pressure source of the microinjector, and filled with cell culture medium. 20 μL of the prepared cell solution was transferred to the culture dish. The pressure was set to 54 Pa to trap the cells into the designed channels. After 10 min, the pressure was reduced and maintained at 7.2 Pa for cell immobilization during injection. The untrapped cells were gently flushed out of the cell holder chip by transfer pipette.

Synthetic modified mRNA (modRNA, StemMAS^TM^) encoding nuclear eGFP was diluted to different concentrations with ultra-pure water (Gibco). The diluted mRNA was centrifuged at 150 × *g* for 15 min, and 1 μL of the supernatant was used to backfill the micropipette with a microloader (Eppendorf). Then the micropipette was mounted on the z-axis of the robot and manually inserted into the cell holder chip.

Injection was performed by the automated microinjection system. The system utilized template matching to recognize and locate cell positions, and the injection motion was controlled by a PID algorithm with visual-based position feedback. As the cells were patterned to predefined positions in an array, the injection motion was simplified and the injection throughput increased. About 420 fL of modRNA was injected into the cell by controlling the injection pressure and injection time. For each injection experiment, about 200 cells were trapped on the cell holder chip and injected. Typically, the whole injection process including the cell loading procedure and injecting cell took about 1 h. After injection, the cells were maintained in the cell holder chip. The cell holder chip with injected cells was placed in a new culture dish with fresh culture medium, and eGFP signals were inspected 18 h after injection with an epi-fluorescence microscope equipped with the appropriate light source and filter sets. The transfection efficiency was determined by calculating the ratio of the number of nuclear eGFP positive cell after 18 h of incubation to the number of the injected cells.

Cell viability was investigated by incubating the cells in culture medium with 0.5 μM of SYTOX Orange dye (Molecular Probes) for 10 min. SYTOX Orange dye could easily penetrate apoptotic cells with compromised plasma membranes, but would not penetrate healthy cell membranes. Cell viability after trapping and injection were evaluated.

The injected cells could be isolated after microinjection. After cell injection, the cell holder chip was rinsed with PBS and put into a new cell culture dish. The trapped cells were released from cell trapping channels by supplying culture medium from chip outlet with positive pressure. Then, the cells were collected with culture medium from chip inlet using a liquid transferring pipette. Supplementary Fig. 5a shows a schematic of the cell retrieving process, and Fig. 5b shows the injected HFF cells retrieved from the cell holder chip and transferred to a glass bottom dish (MatTek Corporation) imaged after 1 day of culture.

### Plasmid cocktail injection

Plasmids encoding eGFP and encoding mCherry were separately mixed at ratios of 1:5, 1:1, 5:1, and 9:1 to a final concentration of 5 ng/μL. The cell preparation and injection process was identical to that described for modRNA injection. Fluorescence signals were examined 48 h after injection. Successfully transfected cells were expected to present red and green fluorescence simultaneously. The transfection efficiency was determined by calculating the ratio of the number of fluorescence labeled cell after 48 h of incubation to the number of the injected cells.

### Imaging and measurement

Cell samples were imaged on a Zeiss Axio Vert.A1 microscope equipped with a CMOS camera (Axiocam 105 color). A mercury arc lamp (HBO 50) was used as the light source with the appropriate set of filter for the excitation and emission wavelengths. Fluorescence Images were acquired by AxioVision software and analyzed with ImageJ software.

### Statistical analysis

All data are reported as mean ± standard deviation (SD) from at least three independent experiments. All statistical analyses were carried out with the Microsoft Excel software.

## Additional Information

**How to cite this article**: Chow, Y. T. *et al.* Single Cell Transfection through Precise Microinjection with Quantitatively Controlled Injection Volumes. *Sci. Rep.* **6**, 24127; doi: 10.1038/srep24127 (2016).

## Supplementary Material

Supplementary Information
