# Supplementary material for: Single Cell Transfection through Precise Microinjection with Quantitatively Controlled Injection Volumes
Source: Sci Rep. 2016 Apr 12;6:24127. doi: 10.1038/srep24127 (PMC4828701; doi:10.1038/srep24127)
Supplement: Supplementary Information [file srep24127-s1.pdf]

Single Cell Transfection through Precise Microinjection with Quantitatively Controlled Injection Volumes

Yu Ting Chow[1,†], Shuxun Chen[1,†], Ran Wang[1], Chichi Liu[2], Chi-wing Kong[3], Ronald A. Li[3,*], Shuk Han Cheng[2,*] and Dong Sun[1,*]

Supplementary Figures

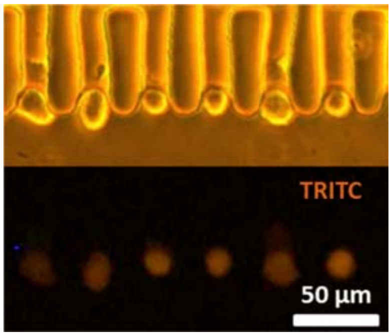

Supplementary Figure 1. Quantitative microinjection on HFF cells. (a) HFF cell injected with TRITC-dextran. (b) The fluorescence intensity of water droplets and cells after injection of the same amount of TRITC-dextran. ($n_{droplet}$ = 10 and n_{cell} = 49)

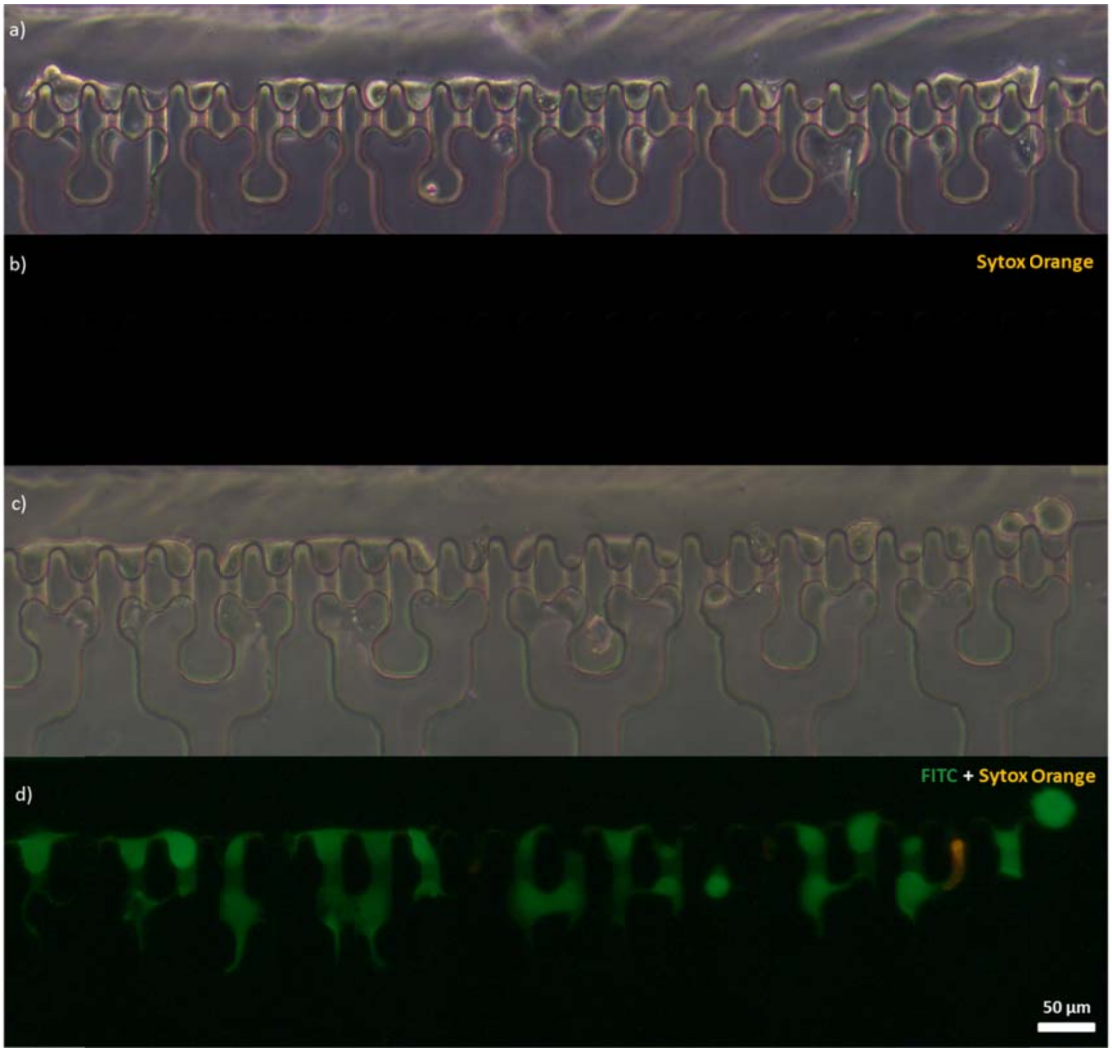

Supplementary Figure 2. The Cell Viability after Trapping and Injection. (a) and (b) Cell viability indicated by SYTOX Orange 1 h after cell trapping. 97.5%±2.0% (mean ± SD, n = 3) trapped cells were viable and adhered to cell holder chip. (c) and (d) Cell viability indicated by SYTOX Orange 1 h after cell injection. An apoptotic cell was positively stained with SYTOX Orange dye. 82.1%±7.0% (mean ± SD, n = 3) of dye injected cells was viable as indicated by SYTOX Orange.

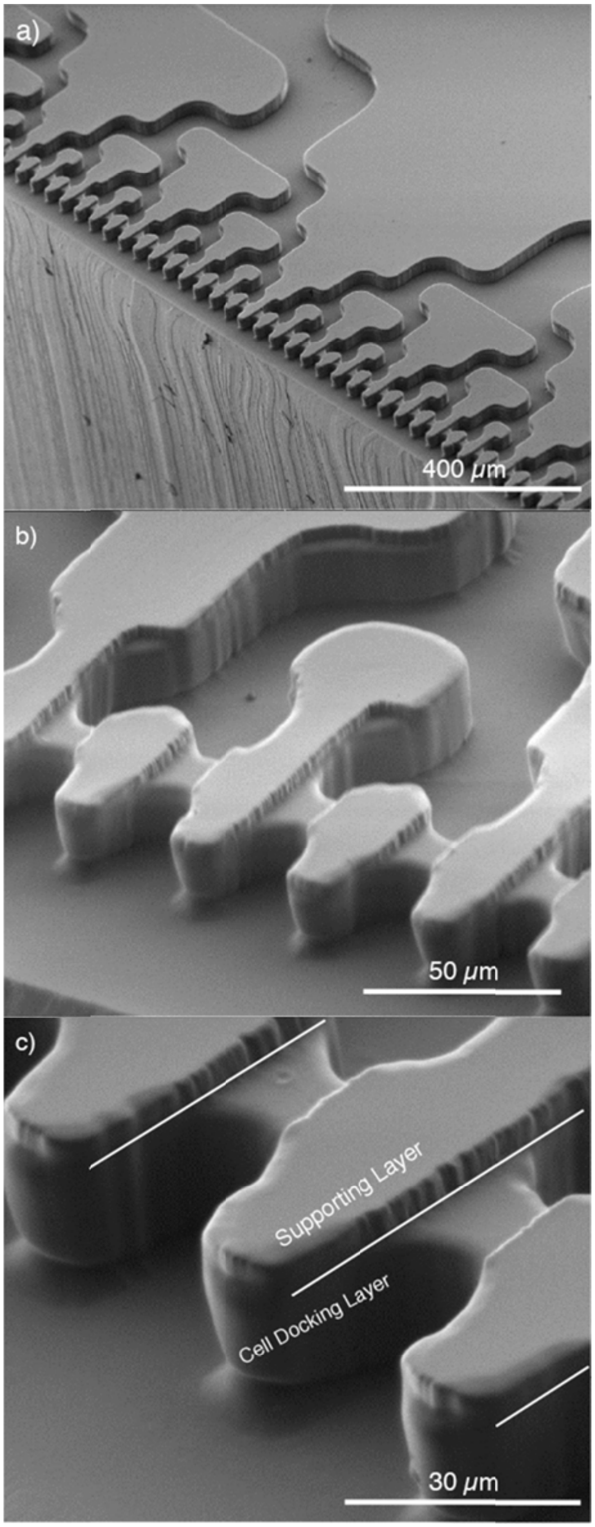

Supplementary Figure 3. Image of the cell trapping channels on cell holder chip by scanning electron microscopy.

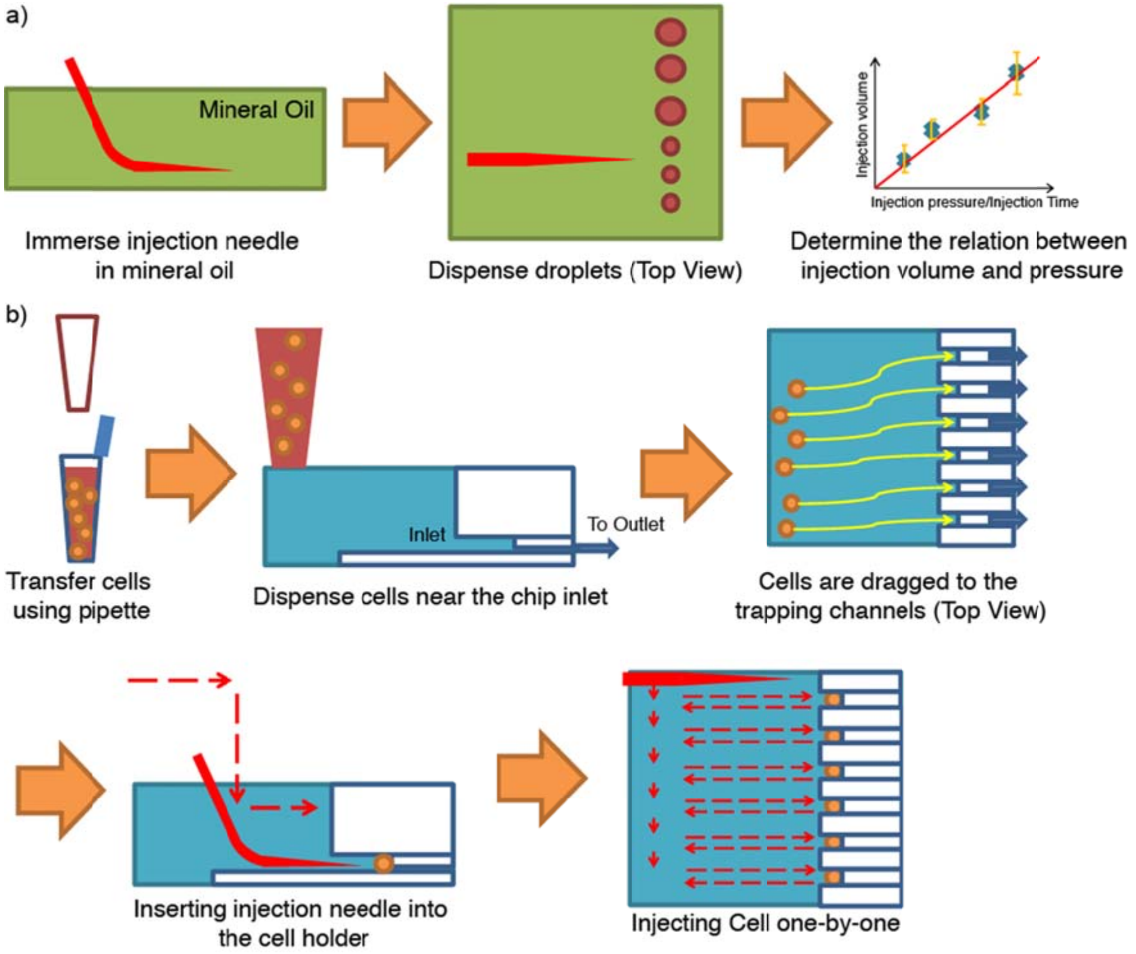

Supplementary Figure 4. Schematic of the calibrated automated cell injection system. (a) The work flow of the injection needle calibration. (b) The work flow of the automated injection system.

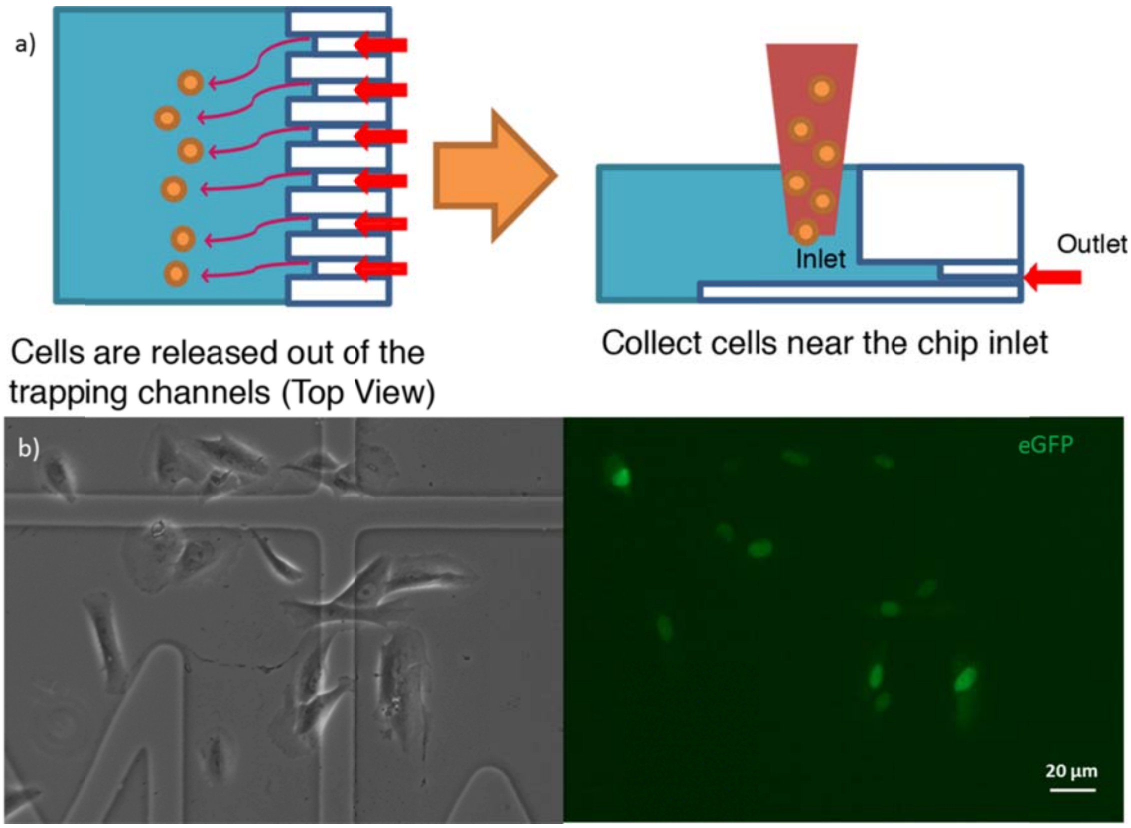

Supplementary Figure 5. **The Retrieval of Injected Cell**. (a) The schematic diagram of the cell retrieval process. After the cell injection process, positive pressure is applied to the outlet of the cell holder. Then, the trapped cells are flushed out by the current. The cells were then collected by pipetting the solution near the inlet. (b) The retrieved HFF cells after 1 day of incubation. The cells were injected with 100 ng/µl nuclear eGFP modRNA.
